# Finding and analysing the minimum set of driver nodes required to control multilayer networks

**DOI:** 10.1038/s41598-018-37046-z

**Published:** 2019-01-24

**Authors:** Jose C. Nacher, Masayuki Ishitsuka, Shuichi Miyazaki, Tatsuya Akutsu

**Affiliations:** 1 Department of Information Science, Toho University, Funabashi, Chiba 274-8510 Japan; 2 Academic Center for Computing Media Studies, Kyoto University, Kyoto, 606-8501 Japan; 3 Bioinformatics Center, Institute for Chemical Research, Kyoto University, Uji, 611-0011 Japan

## Abstract

It is difficult to control multilayer networks in situations with real-world complexity. Here, we first define the multilayer control problem in terms of the minimum dominating set (MDS) controllability framework and mathematically demonstrate that simple formulas can be used to estimate the size of the minimum dominating set in multilayer (MDSM) complex networks. Second, we develop a new algorithm that efficiently identifies the MDSM in up to 6 layers, with several thousand nodes in each layer network. Interestingly, the findings reveal that the MDSM size for similar networks does not significantly differ from that required to control a single network. This result opens future directions for controlling, for example, multiple species by identifying a common set of enzymes or proteins for drug targeting. We apply our methods to 70 genome-wide metabolic networks across major plant lineages, unveiling some relationships between controllability in multilayer networks and metabolic functions at the genome scale.

## Introduction

In recent years, structural controllability and control theory approaches have been studied in depth in the context of linear and nonlinear complex systems and networks^1–4^. More recently, Liu *et al*. mapped the structural controllability problem into one that involves solving a maximum matching (MM) problem^5^. This approach has been used in several studies to investigate control features under different topologies^6,7^. One of the focuses in these studies is to analyze the number of driver nodes, where a set of nodes is called a set of driver nodes if the whole system can be driven from a given initial state to a given target state by applying control signals to only these nodes (see^5^ for the details of the relationship between driver nodes and controllability). However, a large fraction of real-life processes and systems can be better represented by multilayer network structures than by single-layer networks^8,9^. Some groups have extended the MM framework and proposed others to investigate the controllability of multilayer networks. Yuan *et al*. studied the minimum number of driver nodes needed to fully control diffusion dynamics by using matrix computation, where controllers can interact with any layer^10^. Zhang *et al*. studied the controllable subspace of multilayer linear networks, where control signals are applied to only one layer^11^. Pósfai *et al*. studied the controllability of multilayer linear discrete systems with time delays, where control signals are also applied to only one layer^12^. They showed that the minimum number of driver nodes for such systems can be computed based on the maximum network flow. These approaches are useful for controlling multilayer networks in which different layers are connected by some dynamics.

However, there exist other situations in which multiple networks should be independently controlled using the same set of driver nodes. For example, consider the case of controlling biological systems. In such a case, there may exist some differences among networks according to individual differences or species differences, but we need to give the same set of controllers (e.g., the same drugs) or choose one from several sets of controllers. Clearly, for this type of approach, we do not need that these network layers are physically coupled. Menichetti *et al*. studied such type of multilayer network control^13^ by extending the framework of the linear structural controllability of single-layer networks using the maximum matching approach^5^.

Here, we use the minimum dominating set (MDS) approach^14^ to investigate the controllability of multilayer networks instead of using the MM model. A set of nodes in an undirected network is called a dominating set if each node in the network belongs to the set or has a neighbour in the set. A dominating set with the minimum number of elements is called a minimum dominating set (MDS). It was shown in^14^ that if every edge in a network is bi-directional and every node in a dominating set can control itself and all its outgoing edges individually, then the network is structurally controllable by selecting the nodes in a dominating set as the driver nodes. Therefore, an MDS corresponds to a minimum set of driver nodes under this framework. Since the underlying assumptions are different, there is no technical contradiction between both models. However, there are several reasons that motivated us to use the MDS model instead of the MM approach. First, while the MM approach can only guarantee its controllability for linear systems, which are not common in real problems, the MDS model can be applied to nonlinear systems^15^, which exist in abundance in natural problems, because each node has at least one independent control input. Moreover, although the Feedback Vertex Set (FVS)-based control model can also handle non-linear systems, the target states are limited to steady states (including periodic ones)^4,16^. Second, although the MDS is an NP-hard problem, current integer linear programming solver and graph-reduction-based algorithms allow us to find an MDS for very large networks. We will see later that this is the case of the multilayer MDS (MDSM) problem. Third, for values of the power-law degree exponent close to *γ* = 2, the number of driver nodes identified using an MDS is much smaller than that using the MM approach^14^. Finally, the MDS approach has also been adopted by many different groups^17–19^; multiple biological systems have been studied using MDS, such as protein-protein interaction networks^17,20–23^, drug-target networks^15^, ncRNA-protein networks^24^, drug-disease networks^25^ and metabolic networks^26^; and relevant biological findings have been uncovered, such as the enrichment of cancer-related and virus-target genes within the MDS in protein networks^21^. Menichetti *et al*. extensively studied the distribution of the minimum number of driver nodes^13^ based on linear structural controllability^5^; however, no studies on MDS-based controllability using multilayer networks have been conducted.

Here, we first mathematically provide new insights into the MDS controllability framework when multilayer networks are considered. Although the MDS is generally an NP-hard problem, we demonstrate that even in special cases of networks in which the MDS is solved in polynomial time, the multilayer MDS (MDSM) problem is still NP-hard. More importantly, by using a recursive probabilistic technique, we demonstrate that simple formulas can be used to estimate the MDSM size for *k*-regular random networks and maximally assortative scale-free networks. To our knowledge, these are the first results of giving simple formulas to estimate the number of driver nodes in multilayer networks. We also demonstrate that the size of the MDSM does not increase by much compared with that of the MDS for a single network if the difference among the layers in multilayer networks is small.

In spite of the NP-hardness of the multilayer MDS problem, we propose a new algorithm that efficiently computes an MDSM and is able to identify controllers in large-scale multilayer real-world networks. This method includes a novel preprocessing technique based on integer linear programming (ILP), which is our second main result. The algorithm is able to efficiently compute networks of up to 6 layers, with several thousand nodes in each network. Using this novel algorithm, we explore for the first time the optimal solution for the MDSM-based multilayer controllability problem for large genome-wide metabolic networks across major plant lineages. We also analytically and empirically show that the size of the MDSM tends to be close to that of the MDS when the two networks are similar. Finally—but most importantly—we validate the biological importance of the set of nodes included in the MDSMs by compiling data from 70 plant metabolic networks and computing the corresponding MDS and MDSM. The enrichments of the MDS and MDSM in each main metabolic pathway unveil for the first time a relationship between controllability in multilayer networks and metabolic functions at the genome scale.

## Theoretical Results

**Figure 1:**
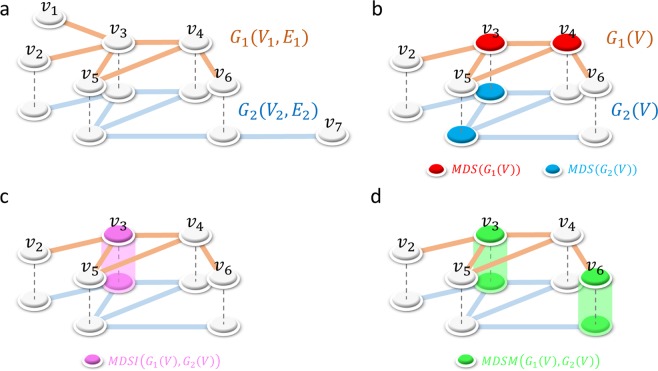
(**a**) A multilayer network defined by graphs *G*_1_(*V*_1_, *E*_1_) and *G*_2_(*V*_2_, *E*_2_). (**b**) The common nodes are defined as $V={V}_{1}\cap {V}_{2}$. From each graph *G*_1_ and *G*_2_, we construct the graphs *G*_1_(*V*) and *G*_2_(*V*) induced by the set of nodes *V*. Then, we compute *MDS*(*G*_1_(*V*)) and *MDS*(*G*_2_(*V*)). (**c**) The MDSI is defined as $MDS({G}_{1}(V))\cap MDS({G}_{2}(V))$. (**d**) The set of nodes (two nodes) that simultaneously multicontrol both layers is defined as *MDSM*(*G*_1_(*V*), *G*_2_(*V*)). Note that although the MDSM is defined for any pair of graphs, $V={V}_{1}\cap {V}_{2}$ is considered in the analysis of metabolic networks. Note that the network layers are not physically coupled.

In this work, we investigate the controllability of multilayer networks using a minimum dominating set (MDS)^14^ approach and focus on a generic type of multilayer network constructed in a manner similar to that of multiplex networks (see Fig. 1). While the definition of a multiplex network states that, in each layer, the same set of nodes is connected by a different set of links^9,27^, here, we allow each layer to consist of a different set of nodes. In other words, the sets of nodes in the multiple layers do not necessarily overlap completely. By considering similar type of networks, such as metabolic networks, a large fraction of nodes are overlapped. Moreover, the network layers are not physically coupled. This situation is often seen in molecular networks, for example, when comparing networks from different organisms that do not synthesize the same set of proteins or enzymes. Concepts closely related to multiplex networks have emerged rapidly and have led to a number of analytical and theoretical developments such as networks of networks, multidimensional networks, multilevel networks and interdependent networks, among others^8,27,28^. In the following, we present the main theoretical findings.

### Upper bound estimation for the size of the MDSM

Our main theoretical result is the development of methods for upper bound estimation of the size of the MDS for multilayer networks (i.e., the size of the MDSM).

Recall that for a graph *G*(*V*, *E*), *U* ($U\subseteq V$) is called a *dominating set* (DS) of *G* if

$$(\forall v\in V)(v\in U\vee (\exists u\in U)(\{u,v\}\in E))$$

holds. A DS with the minimum cardinality is called a *minimum dominating set* (MDS).

MDSM is defined by extending this definition of MDS. Let ${\mathscr{G}}=\{{G}_{i}({V}_{i},{E}_{i})|i=1,\ldots ,N\}$ be a set (multiset) of undirected networks. That is, ${\mathscr{G}}$ corresponds to multilayer networks. Let $V={\cup }_{i\in \{1,\ldots ,N\}}\,{V}_{i}$. *U* ($U\subseteq V$) is called a *dominating set for multilayer networks* ${\mathscr{G}}$ (DSM for ${\mathscr{G}}$) if

$$(\forall i\in \{1,\ldots ,N\})(\forall v\in {V}_{i})(v\in U\vee (\exists u\in U)(\{u,v\}\in {E}_{i}))$$

holds. A DSM with the minimum cardinality is called a *minimum dominating set for multilayer networks* (MDSM). Since an MDSM is also a DS for each *G*_*i*_, if we select an MDSM as a set of driver nodes (with assuming that each driver node can control its links independently), every *G*_*i*_ becomes structurally controllable from the results in^14,15^.

Next, we show a simple property. Let *V*_*MDS*_(*G*_*i*_) be an MDS for *G*_*i*_. Then, *S*_*MDS*_(*G*_*i*_) denotes the MDS size for *G*_*i*_ (i.e., *S*_*MDS*_(*G*_*i*_) = |*V*_*MDS*_(*G*_*i*_)|). Let ${V}_{MDS}({\mathscr{G}})$ and ${S}_{MDS}({\mathscr{G}})$ denote an MDSM and its size for ${\mathscr{G}}$, respectively. Clearly, ${S}_{MDS}({\mathscr{G}})\ge {S}_{MDS}({G}_{i})$ holds for each *G*_*i*_ because an MDSM is also a DS for *G*_*i*_. Conversely, ${\cup }_{i\in \{1,\ldots ,N\}}\,{V}_{MDS}({G}_{i})$ becomes a DSM for ${\mathscr{G}}$. Therefore, we have the following.

**Proposition 1**

$${max}_{i\in \{1,\ldots ,N\}}\,\{{S}_{MDS}({G}_{i})\}\le {S}_{MDS}({\mathscr{G}})\le {\sum }_{i=1}^{N}\,{S}_{MDS}({G}_{i}).$$

Although this result is fairly obvious, it gives strict bounds in the worst case: (i) if *G*_*i*_s are identical, ${S}_{MDS}({G}_{i})={S}_{MDS}({\mathscr{G}})$ holds for all *G*_*i*_; (ii) if *V*_*i*_s are disjoint, ${S}_{MDS}({\mathscr{G}})={\sum }_{i=1}^{N}\,{S}_{MDS}({G}_{i})$ holds. This fact suggests that we should consider a special family of networks. Furthermore, as will be discussed later, the result on artificial networks suggests that the size of the MDSM for *k*-regular random networks is much smaller than this upper bound. Therefore, we theoretically explain this empirical finding.

We assume that graphs are given uniformly at random on the same set of nodes *V* with |*V*| = *n* under the constraint that every node has degree *k*, with *k* being constant. Since a lot of theoretical studies have been done on these *k*-regular random graphs^29^, it is reasonable to consider *k*-regular random graphs in order to investigate theoretical properties of an MDSM.

We utilize the recursive probabilistic estimation technique that was recently developed for analysis of the size of an MDS in *k*-regular random graphs^30^, although our analysis needs additional ideas. It is shown in^30^ that this technique yields very accurate estimates of the MDS size for random graphs. It is to be noted that this technique does not offer rigorous analysis methods but rather approximate analysis methods, as presented in many studies on complex networks using mean-field approximation, cavity methods, and so on.

Let *G*(*U*) denote the subgraph of *G* that is induced by a set of vertices *U*, and let *N*_*G*_(*U*) denote the set of neighbours of *U* in *G* excluding *U* (i.e., ${N}_{G}(U)=\{v|\{u,v\}\in E,u\in U,v\notin U\}$). We consider the following virtual procedure that outputs a DSM for ${\mathscr{G}}=\{{G}_{1},\ldots ,{G}_{N}\}$.

(i) Let *DS*_1_ be the dominating set for *G*_1_ obtained using the method in^30^. Let ${V}_{1}\leftarrow V$.
(ii) For *i* = 2 to *N*, perform steps (iii)–(iv).
(iii) Let ${V}_{i}\leftarrow V-\underset{j=1}{\overset{i-1}{\cup }}\,D{S}_{j}-{N}_{{G}_{i}}(\underset{j=1}{\overset{i-1}{\cup }}\,D{S}_{j})$. (*V*_*i*_ is the set of vertices in *G*_*i*_ that are not dominated by a combined dominating set for *G*_1_, …, *G*_*i*−1_).
(iv) Let *DS*_*i*_ be the dominating set for *G*_*i*_(*V*_*i*_), obtained using the method in^30^.
(v) Output $D{S}_{1}\cup D{S}_{2}\cup \cdots \cup D{S}_{N}$ as a DSM.

It is obvious that this procedure outputs a correct DSM (but not necessarily an MDSM) for ${\mathscr{G}}$. By analysing this procedure, the size of the resulting DSM is estimated as *α*_*N*_*n*, where *α*_*i*_ is given by

$$\begin{array}{rcl}{\alpha }_{1} & = & \frac{1}{k+1},\\ {\alpha }_{i+1} & = & {\alpha }_{i}+\frac{1}{k+1}{(1-{\alpha }_{i})}^{k+1}.\end{array}$$

Although it only gives an estimate of the upper bound of the size of MDSM, comparison with the computational results (see Supplementary Information (SI)) suggests that this simple formula (for an upper bound) gives a very accurate estimate of the MDSM size for *k*-regular random networks. This estimation method can be modified for the analysis of maximally assortative scale-free networks in which the degree distribution follows a power law $\propto {k}^{-\gamma }$, where it is known that many real-world networks have both scale-free and assortative properties^30^.

A network is called *maximally assortative* if an exchange of any pair of edges does not increase the assortative coefficient (see SI). It is shown in^30^ that a maximally assortative network is approximately regarded as a collection of *k*-regular networks. By using the same virtual procedure as presented above, the size of the resulting DSM is estimated as *β*_*N*_*n*, where *β*_*i*_ is given by

$$\begin{array}{rcl}{\beta }_{1} & = & \frac{{\sum }_{k=1}^{\infty }\,\frac{1}{k+1}\cdot {k}^{-\gamma }}{{\sum }_{k=1}^{\infty }\,{k}^{-\gamma }},\\ {\beta }_{i+1} & = & {\beta }_{i}+(\frac{{\sum }_{k=1}^{\infty }\,(\frac{1}{k+1}){(1-{\beta }_{i})}^{k+1}{k}^{-\gamma }}{{\sum }_{k=1}^{\infty }\,{k}^{-\gamma }}).\end{array}$$

Comparison with the computational results (see SI) suggests that this formula gives a reasonable estimate of the MDSM size for maximally assortative scale-free networks.

Upper bound estimations and the result on artificial networks suggest that the MDSM size converges to *n* as the number of networks grows. We show below that this speculation is true (see SI for the proof).

**Proposition 2**

Suppose that each graph in multilayer networks has the minimum degree *d*_*min*_. Then, the MDSM size is at least *n* − *d*_*min*_ if a sufficient number of distinct graphs are given and *n* is sufficiently large.

### Hardness of computation of MDSM

It is known that the maximum bipartite matching for networks of up to two layers can be determined in polynomial time, whereas the computation of such a matching for networks of three or more layers is NP-hard^31^. This fact suggests that the minimum set of driver nodes under linear structural controllability^5^ can be obtained in polynomial time only for networks of up to two layers.

On the other hand, it is known that the computation of an MDS is NP-hard even for one network^32^. However, the situation changes if we consider special graph classes: it is known that an MDS can be computed in polynomial time if a given network is a partial *k*-tree (for a constant *k*)^32^. As a special case, it is seen that an MDS can be computed in polynomial time if networks are forests or consist of cycles and stars. We can show that the computation of an MDSM is NP-hard even for such simple networks (see SI for the proofs).

#### Theorem 3

The MDSM problem for two-layer networks is NP-hard even if a graph in each layer consists of cycles and at most one star.

#### Theorem 4

The MDSM problem for three-layer networks is NP-hard even if a graph in each layer does not contain any cycle.

These theorems suggest that the use of multilayers causes easy cases to be difficult.

### Results on artificial networks

We performed numerical experiments to examine how the MDSM size changes as the number of wiring operations increases. Note that in this section, we consider similar networks, different from the Theoretical Results section. We employed the FAST-MDSM method mentioned in the Methods section using the SCIP ILP solver (http://scip.zib.de/).

We generated random graphs that follow the power-law degree distribution *k*^−*γ*^, with each containing approximately *n* = 5,000 nodes. For each generated graph *G*_0_, we applied *K* rewiring operations and obtained a graph *G*_*K*_ (see Methods and Fig. S1). Then, we computed *S*_*MDS*_(*G*_0_, *G*_*K*_) and *S*_*MDS*_(*G*_0_) by using FAST-MDSM, where computation of an MDSM was completed in a few seconds per graph pair on a PC with an Intel Core (TM) i7-3517U CPU and 4 GB of RAM.

For each *γ* = 2.1, 2.3, 2.5, 2.7, we calculated the average ratio of *S*_*MDS*_(*G*_0_, *G*_*K*_) (obtained using FAST-MDSM) to *S*_*MDS*_(*G*_0_) over 10 trials. The averaged results over 10 trials are shown in Fig. S2, from which it can be seen that the increase in the size of the MDSM is much smaller than *K*. In addition, we compared these ratios with those obtained via the theoretical estimate described in the Methods section. The results are shown in Fig. S3(a). We can see that the actual ratio does not linearly increase as *N* increases, whereas the theoretically estimated ratio increases almost linearly as *N* grows. We can also see that there is substantial discrepancy between the ratios obtained using FAST-MDS and those obtained using the theoretical estimate—especially for large *K*. This discrepancy is reasonable because the rewiring operations do not change the degree of each node and, thus, high degree nodes tend to remain in the MDSM.

We also compared the ratios obtained using FAST-MDSM with those obtained using the theoretical estimate described in the Methods section for the case of non-degree preservation modifications (i.e., insertion and deletions of random edges). The results are shown in Fig. S3(b), where the average ratio over 10 trials is shown for each case. We can see a good agreement between the simulation results and the theoretical estimates for the cases of a non-large number of insertions and deletions of random edges. The reason for the much better agreement may be that the degree is not necessarily preserved in this case.

### Results on real networks

To examine the usefulness of the MDSM for the analysis of biological networks, we applied the MDSM to two kinds of large-scale multilayer biological networks: plant metabolic networks and protein-protein interaction networks.

**Figure 2:**
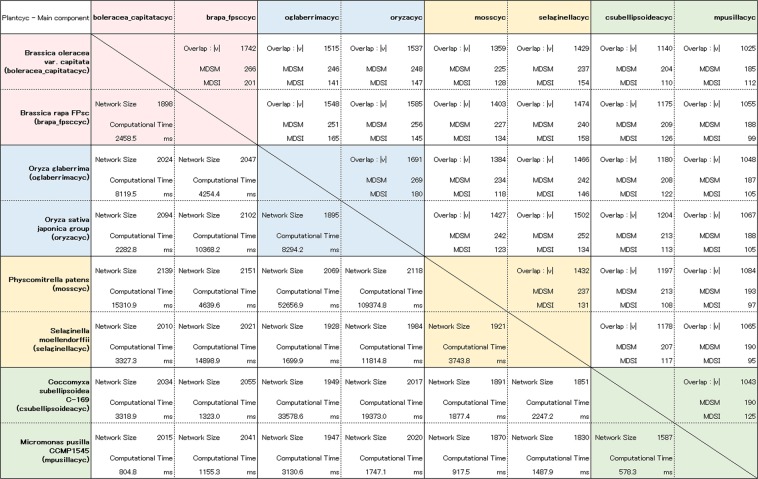
A representative set of eight metabolic networks from the angiosperm plant lineage, including eudicot and monocot subgroups and the early land plant and green algae lineages. (Upper matrix) The number of common enzymes between both species is denoted as |*V*|. MDSM and MDSI numbers are indicated in the box cells. (Lower matrix) The network size and the computational time (milliseconds) required to execute the FAST-MDSM method are shown. The total number of nodes and the MDS for each single network are shown in the first column.

**Figure 3:**
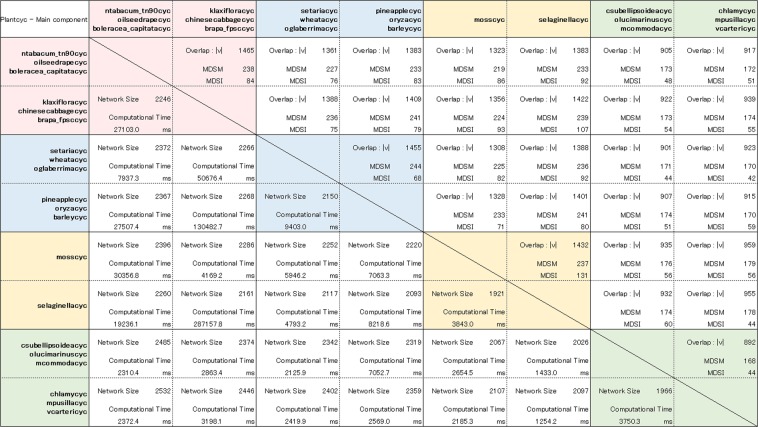
Same as Fig. 2 but considering a 6-layer network analysis in which each group consists of up to three species. The results for the complete set of 70 metabolic networks for the 2-layer analysis and for the set of 25 groups for the 6-layer analysis are shown in the Supplementary Information file (Tables S1 and S2, respectively).

First, we applied the developed fast method of computing the MDS for multilayer networks (FAST-MDSM) to the study of genome-wide metabolic networks across major plant lineages compiled from the Plant Metabolic Network database 16.0^33,34^. To validate the biological relevance and the functional role of the nodes (enzymes) included in the MDSMs, we performed a comparative analysis of plant metabolic networks. We collected 70 species of metabolic networks corresponding to major plant lineagesâ€” from green algae and early land plants to angiosperms. The angiosperm lineage was further subdivided into major evolutionary groups: monocots (such as grasses and cereals) and eudicots. We then computed the corresponding MDSM for each pair of networks. The resulting MDSM identifies the common minimum number of enzymes that must be simultaneously controlled in both networks. To compare these with a case involving a simple overlap between networks, we consider the MDSI (minimum dominating set induced) defined in the Methods section and illustrated in Fig. 1. Note that the MDSI is a simple intersection of the MDS; therefore, its controllability role is unclear, which highlights the novelty and importance of the MDSM. The results shown in Fig. 2 (upper matrix) correspond to eight species and indicate that the size of the MDSM tends to be closer to that of the MDSI when the two networks belong to the same evolutionary group. On the other hand, Fig. 2 (lower matrix) shows the network size and the computational time in milliseconds required to execute the FAST-MDSM method using two-layer networks. See the SI files for details of all 70 species (Table S1). To illustrate the efficiency of the proposed algorithm, we also considered multilayer network analysis with more than two networks. The results for a 6-layer network analysis, in which one group is composed of up to three species, are shown in Fig. 3. We see that the computational time is the same order of magnitude of that necessary to compute a much simpler 2-layer network in many cases. The results also show that this multilayer network problem can be quickly solved using the proposed method, in spite of the fact that the MDS is an NP-hard problem (see Fig. 3 (lower matrix)). See also the SI for the complete results of the 6-layer network analysis involving 25 groups (Table S2).

Next, to verify the algorithm performance for even larger real-world networks, we downloaded protein-protein interaction networks from the HINT database version 4.0. The results in Fig. S4 (upper matrix) show that the size of the MDSM is still close to that of the MDSI (see *H*. *sapiens* vs. *D*. *Melanogaster* results in Fig. S4). This finding illustrates the advantages of controlling several networks simultaneously because the identified MDSM set does not significantly differ from the set required to control a single network in many cases. The results show that these large networks can be quickly solved using the proposed method, in spite of the fact that the MDS is an NP-hard problem (see Fig. S4, lower matrix). The results for 3-layer, 4-layer PPI networks are shown in the SI (Table S3).

**Figure 4:**
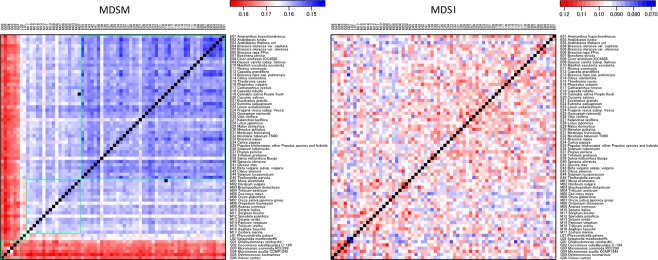
The heat map of the fraction of MDSM (left) and MDSI (right) for each pair of metabolic networks from green algae (G), early land plants (L), eudicots (E) and monocots (M). This result highlights the fact that the MDSMs cluster according to each major plant lineage (see green box). The MDSM size tends to be smaller within each lineage, facilitating the control of each network pair in each major group. The analysis involves 70 species.

**Figure 5:**
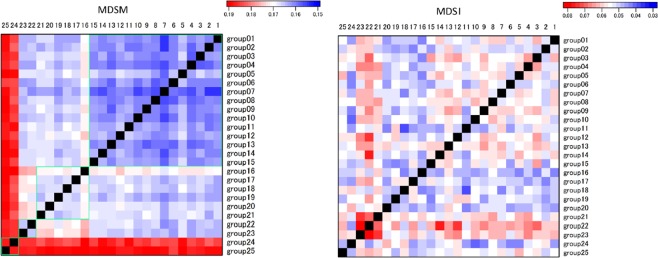
Same as Fig. 4 but for a 6-layer network analysis in which 25 groups consisting of up to 3 layers were analysed. This result highlights the fact that the MDSMs cluster according to each major plant lineage (see green box) whereas MDSI does not recognise the plant lineages.

Then, the fractions of the MDSMs and MDSIs were represented in a heat map for all 70 species using a 2-layer network analysis and a 6-layer network analysis in which one group was composed of up to three species (see Figs 4 and 5, respectively). The result highlights the fact that the MDSMs cluster according to each major plant lineage. In other words, species belonging to the same lineage tend to have a higher similarity of reaction node sets^33^; therefore, each MDSM tends to be smaller within each lineage, facilitating the control of each network pair.

It is worth considering the multilayer network problem as a single network. Therefore, we compute an MDS for the union of two graphs *G*_1_ and *G*_2_, instead of considering a two-layer network analysis. Two graphs can be combined (${G}_{1}\cup {G}_{2}$) as shown in Fig. S7. Then, the MDS results show that the resulting union graph does not increase notably the required MDS size to control the network. Specially, when both networks belong to the same lineage, the MDS gives similar results (see Fig. S8).

**Figure 6:**
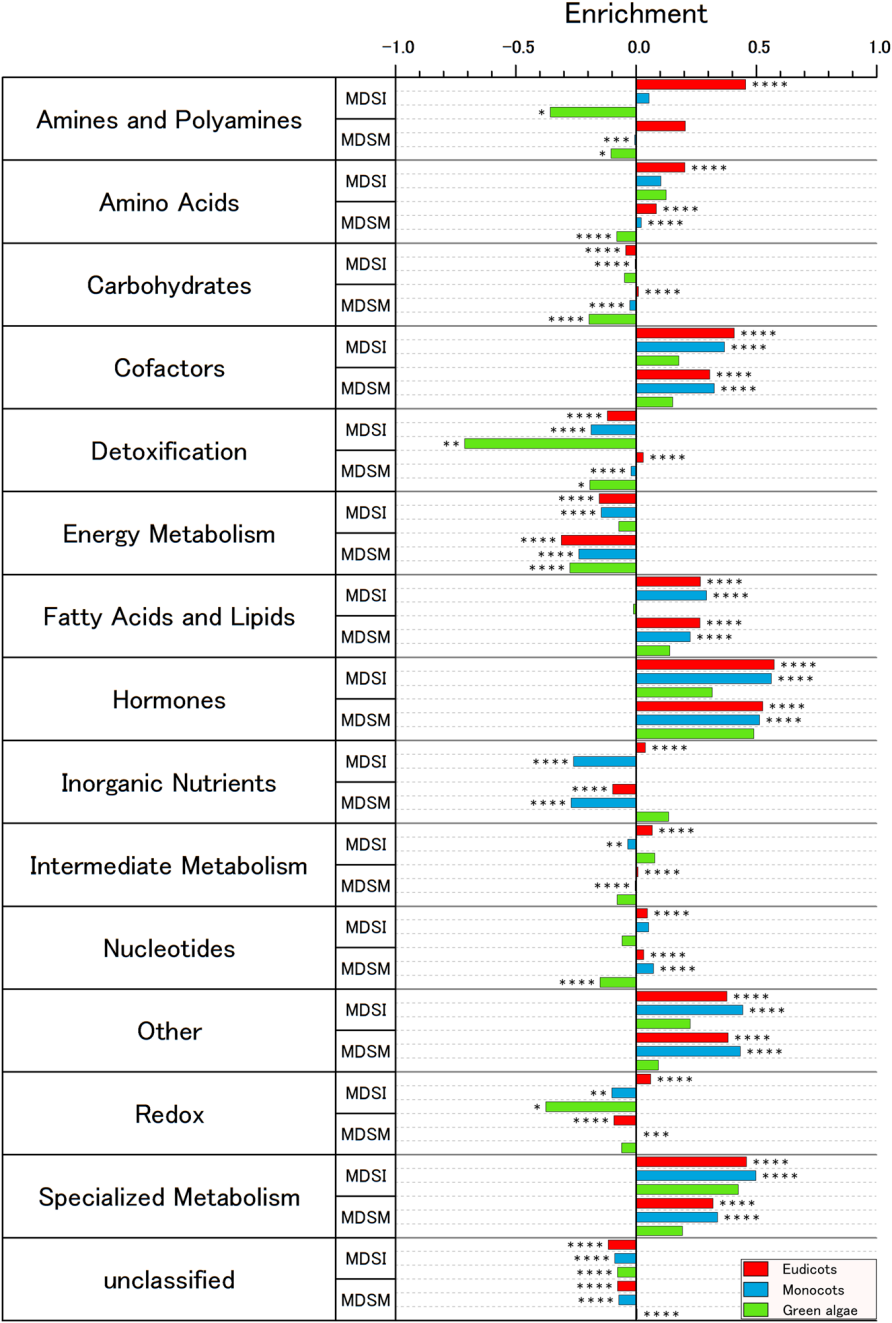
The average enrichment of the functional classes of metabolic reactions classified for each major lineage group. The results suggest that the functional classes of amino acids and specialized metabolism show a tendency to benefit from network multicontrol via MDSM rather than via MDSI. In contrast, the results for the functional classes of fatty acids and lipids and hormone metabolism show an enhanced tendency to benefit from MDSI control. The statistical analysis results are denoted as follows: ****p-value < 0.0001, ***p-value < 0.001, **p-value < 0.01 and *p-value < 0.05.

To elucidate the biological functionality and importance of the reaction node sets associated with the MDSM, we computed the average enrichment of the functional classes of the metabolic reactions classified for each plant lineage (green algae, monocots and eudicots) (see Methods and Fig. 6). We excluded early land plants from this analysis because only two samples were available. First, the results show similar control trends for monocots (e.g., grasses and cereals) and eudicots because they share a more similar metabolism than do the algae. In contrast, the differences between angiosperms and algae are more evident. A more detailed analysis was performed using the enrichment results of each functional class and major plant lineage. Fourteen functional classes were used to classify the enzymes and reactions of each species. For example, consider the specialized metabolism functional class. For the MDSM, the absolute difference in enrichment (er) between eudicots (E) and monocots (M) (both are angiosperm major groups) is much smaller than that between eudicots and green algae (A), as follows: $(|er(E)-er(M)|\ll |er(E)-er(A)|)$. In contrast, these two differences have approximately the same strength for the MDSI $(|er(E)-er(M)|\approx |er(E)-er(A)|)$. Therefore, by using these criteria, the results in Fig. 6 suggest that the functional classes of amino acids and specialized metabolism show a tendency to benefit from network multicontrol via MDSM rather than via MDSI. In contrast, the results for the functional classes of fatty acids and lipids and for hormone metabolism show an increased tendency to benefit from MDSI control.

## Discussion

We developed a new FAST-MDS computation that allows us to efficiently identify the minimum driver nodes in large multilayer networks. The application of the developed tools to metabolism in major plant lineages showed that, as expected, for each pair of species, a subset of the functional metabolic classes could be more efficiently multicontrolled using a common subset of enzymes.

In previous studies, the abundance of chemical reactions in functional classes was investigated from an evolutionary perspective for major plant lineages, including early land plants and angiosperms. The results suggested a significant depletion of carbohydrates, amino acids, nucleotides, energy and cofactors metabolism in angiosperms. On the other hand, early plants and angiosperms showed an enrichment of carbohydrates and a specialized metabolism, respectively^33^.

The results shown in Fig. 6 suggest that the specialized metabolism functional class tends to benefit from multicontrol MDSM. This metabolic pathway generates specialized metabolites that are useful for adjusting the cell state to the surroundings and environmental conditions^35^. The secondary metabolism has an inherent plasticity that facilitates its control at different levels. It has been reported that specific gene clusters are responsible for the synthesis of specialized metabolites in bacteria and fungi^36,37^. Studies have shown that the control and modification of these regulatory processes can lead to the production of desired specialized metabolites^38^. A similar approach could be applied to plants; indeed, one transcriptional regulator has already been associated with a plant metabolic gene cluster^39,40^. Our results indicate that in some cases, the control of multiple pathways and species could be even more efficient in terms of the minimum number of required driver enzymes than that required to control specific pathways and species separately.

First, the presented findings suggest future directions for controlling multiple species by identifying a common set of enzymes or genes for regulation or drug targeting. This strategy may be useful in developing drugs that can kill several kinds of harmful bacteria. Second, cellular networks may differ among individuals. However, our computational findings suggest that we may be able to use the same set of drugs for many patients.

In conclusion, the tools and methodologies presented for controlling multilayer networks using a common set of nodes could lead to broad research directions, ranging from evolutionary biology to drug design and development, that deserve further exploration.

## Methods

### Metabolic network analysis

A set of genome-wide plant metabolic networks was compiled from the publicly available Plant Metabolic Network (PMN) database version 16.0^34^. The set included 70 species from major plant lineages: six species correspond to green algae, two species are early land plants, 17 species are monocots, and 45 species are eudicots. The analysis was performed using an enzyme/reaction-centric network assembled from the full metabolic pathways, in which enzymes are considered as control targets. In multiple layers problem, the shared enzymes may potentially lead to identify key enzymes to control multiple organisms. Therefore, we consider the intersection of nodes among layers as shown in Fig. 1. To validate the biological importance of the nodes engaged in the MDSMs, we compared them with the MDSI, which is defined as follows (see also Fig. 1):

1. From two species of metabolic networks defined as *G*_1_(*V*_1_, *E*_1_) and *G*_2_(*V*_2_, *E*_2_), we compute the common nodes defined as $V={V}_{1}\cap {V}_{2}$.
2. From each graph *G*_1_ and *G*_2_, we compute the graphs *G*_1_(*V*) and *G*_2_(*V*) induced by the set of nodes *V*.
3. Then, we compute *MDS*(*G*_1_(*V*)), *MDS*(*G*_2_(*V*)) and *MDSM*(*G*_1_(*V*), *G*_2_(*V*)).
4. Finally, we compare *MDSM*(*G*_1_(*V*), *G*_2_(*V*)) with $MDS({G}_{1}(V))\cap MDS({G}_{2}(V))$. The latter is called the MDSI.

For the above metabolic network analysis, the ILP was determined using the GLPK LP/MIP Solver v4.55. The enrichment of a multicontrol feature *C* (MDSI or MDSM) in a given functional class *F* of enzymes for a pair of networks reads as ${E}^{C}(F)=\,\mathrm{ln}\,[({N}_{p}^{C}(F)/{N}_{p}^{C})/({N}_{p}(F)/{N}_{p})]$, where *N*_*p*_ indicates the total number of enzymes common in both organisms and *N*_*p*_(*F*) indicates the total number of enzymes common in both organisms that also belong to the *F* functional class. ${N}_{p}^{C}$ refers to the total number of enzymes that are in the multicontrol set *C* computed for both organisms (included in *N*_*p*_). ${N}_{p}^{C}(F)$ indicates the total number of enzymes that are in the control set *C* computed for both organisms and that belong to the *F* functional class (included in *N*_*p*_(*F*)).

### FAST-MDSM computational procedure

Although both MDS and MDSM computation are NP-hard, optimal solutions can be obtained for large networks by using a simple integer linear programming (ILP) formulation if networks have scale-free properties. Furthermore, for two-layer networks, MDSMs can be obtained more quickly using the heuristic preprocessing method given below, which has some similarity with the generalized leaf-removal procedure proposed for single-layer networks in^19^ but is substantially different to cope with multilayer networks. In this subsection, we provide ILP-based procedures for both two-layer networks and networks with more than two layers, both of which are referred to as FAST-MDSM.

First, we determine a subset *V*_*M*_ of an MDSM in the following way by focusing on degree 1 nodes, where *v* is *observed* in *G*_*i*_ if *v* has a neighbouring node *v*′ ∈ *V*_*M*_ in *G*_*i*_. Note that ${d}_{{G}_{i}}(v)$ indicates the degree of *v* in *G*_*i*_.

1. Let $${V}_{M}\leftarrow \{\}$$.
2. Repeat steps (3)–(8) until no more nodes are added to *V*_*M*_.
3. For all nodes ${v}_{i}\in {V}_{1}\cup {V}_{2}$, perform steps (4)–(6).
4. If there exists an unobserved ${v}_{j}\notin {V}_{M}$ in *G*_1_ such that ${d}_{{G}_{1}}({v}_{j})=1$, {*v*_*i*_, *v*_*j*_} ∈ *E*_1_ and ${v}_{j}\notin {V}_{2}$ hold, then delete {*v*_*i*_, *v*_*j*_} from *E*_1_, add *v*_*i*_ to *V*_*M*_, and let *v*_*j*_ be observed in *G*_1_.
5. If there exists an unobserved ${v}_{j}\notin {V}_{M}$ in *G*_2_ such that ${d}_{{G}_{2}}({v}_{j})=1$, {*v*_*i*_, *v*_*j*_} ∈ *E*_2_ and ${v}_{j}\notin {V}_{1}$ hold, then delete {*v*_*i*_, *v*_*j*_} from *E*_2_, add *v*_*i*_ to *V*_*M*_, and let *v*_*j*_ be observed in *G*_2_.
6. If there exists an unobserved ${v}_{j}\notin {V}_{M}$ such that ${d}_{{G}_{1}}({v}_{j})=1$, ${d}_{{G}_{2}}({v}_{j})=1$, {*v*_*i*_, *v*_*j*_} ∈ *E*_1_ and {*v*_*i*_, *v*_*j*_} ∈ *E*_2_ hold, then delete {*v*_*i*_, *v*_*j*_} from *E*_1_ and *E*_2_, add *v*_*i*_ to *V*_*M*_, and let *v*_*j*_ be observed in *G*_1_ and *G*_2_.
7. For all unobserved nodes ${v}_{i}\notin {V}_{M}$ in *G*_1_, if there exists *v*_*j*_ ∈ *V*_*M*_ such that {*v*_*i*_, *v*_*j*_} ∈ *E*_1_, let *v*_*i*_ be observed in *G*_1_.
8. For all unobserved nodes ${v}_{i}\notin {V}_{M}$ in *G*_2_, if there exists *v*_*j*_ ∈ *V*_*M*_ such that {*v*_*i*_, *v*_*j*_} ∈ *E*_2_, let *v*_*i*_ be observed in *G*_2_.

It is obvious that this procedure never puts a *v*_*i*_ into a *V*_*M*_ that is not included in an MDSM; thus, it is ensured that *V*_*M*_ is a subset of an MDSM. Then, we apply the following ILP, where each *x*_*i*_ is a binary variable (i.e., *x*_*i*_ takes either 0 or 1).

$$\begin{array}{l}{\rm{minimize}}\,{\sum }_{i=1}^{n}\,{x}_{i},\\ {\rm{subject}}\,{\rm{to}}\,{x}_{i}=1\,{\rm{for}}\,{\rm{all}}\,{v}_{i}\in {V}_{M},\\ \,{x}_{i}+{\sum }_{j:\{{v}_{j},{v}_{i}\}\in {E}_{1}}\,{x}_{j}\ge 1,\,{\rm{for}}\,{\rm{all}}\,{v}_{i}\in {V}_{1}-{V}_{M}\,{\rm{not}}\,{\rm{observed}}\,{\rm{in}}\,{G}_{1},\\ \,{x}_{i}+{\sum }_{j:\{{v}_{j},{v}_{i}\}\in {E}_{2}}\,{x}_{j}\ge 1\,{\rm{for}}\,{\rm{all}}\,{v}_{i}\in {V}_{2}-{V}_{M}\,{\rm{not}}\,{\rm{observed}}\,{\rm{in}}\,{G}_{2}.\end{array}$$

In this ILP, *x*_*i*_ = 1 corresponds to *v*_*i*_ ∈ *V*_*MDSM*_, where *V*_*MDSM*_ is the set of nodes in an MDSM computed using this ILP-based method. The first line states that the number of nodes in *V*_*MDSM*_ must be minimized. The second line states that every *v*_*i*_ ∈ *V*_*M*_ must be included in *V*_*MDSM*_. The third (resp., fourth) line states that every *v*_*i*_ ∈ *V*_1_ − *V*_*M*_ (resp., *v*_*i*_ ∈ *V*_2_ − *V*_*M*_) must be included in *V*_*MDSM*_ (i.e., *x*_*i*_ = 1) or must have a neighbour *v*_*j*_ ∈ *V*_*MDSM*_. Since an ILP solver always outputs an optimal solution (if computation is finished), it is shown that this procedure clearly finds an MDSM.

This preprocessing method can be generalized for networks of three or more layers. However, the following simple ILP formulation works enough well for three or more layer networks as well as for two-layer networks.

$$\begin{array}{l}{\rm{minimize}}\\ {\rm{subject}}\,{\rm{to}}\end{array}\,\begin{array}{c}{\sum }_{i=1}^{n}\,{x}_{i},\\ {x}_{i}+{\sum }_{j:\{{v}_{j},{v}_{i}\}\in {E}_{k}}\,{x}_{j}\ge 1\,{\rm{for}}\,{\rm{all}}\,{v}_{i}\in {V}_{k},\,{\rm{for}}\,{\rm{all}}\,k=1,\ldots ,N.\end{array}$$

There exist two variants in its implementation: (i) ignoring isolated nodes and (ii) not ignoring isolated nodes, because if an isolated node exists, it must be included in an MDSM under the original definition.

As will be shown later, the above ILP-based methods work efficiently for real biological networks. Although exact reasons are very unclear, one possible reason is that most real networks have power-law degree distributions with a low average degree and thus have many degree 1 and degree 2 nodes by which many MDSM nodes are determined based only on the local topology and/or progressively.

### Degree-preserving rewiring

Here, we present a theoretical analysis by employing degree-preserving rewiring, which has been widely used in the study of complex networks, to modify a given network while preserving the degree distribution (see also Fig. S1). In this method, we randomly choose a pair of edges {*v*_*i*_, *v*_*j*_} and {*v*_*h*_, *v*_*k*_} in a given graph *G*(*V*, *E*) such that $\{{v}_{i},{v}_{k}\}\notin E$ and $\{{v}_{h},{v}_{j}\}\notin E$. Then, we delete these edges and add two edges {*v*_*i*_, *v*_*k*_} and {*v*_*h*_, *v*_*j*_}. We repeat this procedure *K* times. Clearly, the degree of each node does not change; thus, the degree distribution is preserved. Let *G*_0_ be the original network and *G*_*K*_ be the network obtained by applying degree-preserving rewiring *K* times.

We here give a quantitative estimate of the difference between *S*_*MDS*_(*G*_0_, *G*_0_) = *S*_*MDS*_(*G*_0_) and *S*_*MDS*_(*G*_0_, *G*_1_). It is expected that ${S}_{MDS}({G}_{0},{G}_{K})\le K\cdot {S}_{MDS}({G}_{0},{G}_{1})$ approximately holds in many cases, although it does not always hold because the size of an MDSM is sensitive to exchanged edges. Let *V*_*MDS*_ be an MDS for *G*_0_(*V*_0_, *E*_0_). We can obtain a dominating set for (*G*_0_, *G*_1_) by adding at most one node to *V*_*MDS*_. Specifically, we may need to add one node to *V*_*MDS*_ only for the following cases (see Fig. S1):

(i) $$|\{{v}_{i},{v}_{j},{v}_{h},{v}_{k}\}\cap {V}_{MDS}|=1$$,
(ii) $$|\{{v}_{i},{v}_{k}\}\cap {V}_{MDS}|=1$$ and $$\{{v}_{j},{v}_{h}\}\cap {V}_{MDS}=\varnothing $$,
(iii) $$|\{{v}_{j},{v}_{h}\}\cap {V}_{MDS}|=1$$ and $$\{{v}_{i},{v}_{k}\}\cap {V}_{MDS}=\varnothing $$.

Let *p* be the probability that an arbitrary endpoint of a randomly chosen edge of *G*_0_ belongs to *V*_*MDS*_, which is estimated as

$$p\approx \frac{{\sum }_{v\in {V}_{MDS}}\,{d}_{{G}_{0}}(v)}{{\sum }_{v\in {V}_{0}}\,{d}_{{G}_{0}}(v)}.$$

Then, case (i) holds with probability 4*p*(1 − *p*)^3^ if there was no constraint on the chosen edges, where ‘4’ comes from the fact that any one of *v*_*i*_, *v*_*j*_, *v*_*h*_, *v*_*k*_ can be a node in an MDS. Both case (ii) and case (iii) hold with probability *p*^2^(1 − *p*)^2^ if there were no constraints on the chosen edges. Here, we should note that in case (i), if *v*_*j*_ has another neighbour in *V*_*MDS*_, then we need not add a node to *V*_*MDS*_. Similarly, we need not add a node in case (ii) (resp., case (iii)) if either *v*_*j*_ or *v*_*h*_ (resp., *v*_*i*_ and *v*_*k*_) has another neighbour in *V*_*MDS*_. Thus, we need to consider the probability *q* that an arbitrary node $v\notin {V}_{MDS}$ has more than one neighbouring node in *V*_*MDS*_, which is given by the ratio of the number of such nodes to the total number of nodes not in *V*_*MDS*_. Therefore, the probability *P*_+1_ that we need to add one node to *V*_*MDS*_ is estimated as

1 $${P}_{+1}\approx 4(1-q)p{(1-p)}^{3}+2{(1-q)}^{2}{p}^{2}{(1-p)}^{2}.$$

If we apply rewiring *K* times, ${S}_{MDS}({G}_{0},{G}_{K})\le K\cdot {P}_{+1}$ would approximately hold for small *K*. In the Results on artificial networks section, we compared this estimate of *S*_*MDS*_(*G*_0_, *G*_*K*_) with its actual size using artificially generated scale-free networks. It is to be noted that although *P*_+1_ is calculated from the empirical distribution of *k*-degree nodes in an MDS, we might be able to obtain an analytical expression of *P*_+1_ if such a distribution could be analytically obtained.

### Random insertions and deletion of edges

Next, we consider the case of the random insertions and deletion of edges. We assume that *G*_*i*+1_ is created from *G*_*i*_ via either edge deletion or edge insertion as follows:

- One edge is randomly selected from *G*_*i*_ and deleted with probability *p*_*d*_;
- Otherwise, a pair of nodes (not connected by an edge) is randomly selected and connected by an edge (with probability 1 − *p*_*d*_).

Since it is difficult to estimate the change in the MDS size due to edge insertions, we focus on edge deletions. Let {*v*_*i*_, *v*_*j*_} be an edge to be deleted from *G*_0_. Suppose that ${v}_{j}\notin {V}_{MDS}$, *v*_*i*_ ∈ *V*_*MDS*_, and *v*_*j*_ does not have more than one neighbouring node in *V*_*MDS*_. Then, *v*_*j*_ must be added to *V*_*MDS*_. The probability of occurrence of such a case is estimated as (1 − *q*)*p*(1 − *p*). Since we need to consider the symmetric case and the probability *p*_*d*_, the probability that one node is added to *V*_*MDS*_ is estimated as

2 $${P}_{+1}\approx 2{p}_{d}(1-q)p(1-p).$$

## Supplementary information

Supplementary Information File

Data For Table S1

Data For Table S2

Data For Table S3

## Data Availability

All data related to metabolic patways analysed in this work were downloaded from the publicly available Plant Metabolic Network Database Version 16.0 (PMN) www.plantcyc.org. Because this is a public database we cannot re-upload the entire datasets in our submission. All the computational results are presented in figures and included in this manuscript and in the Supplementary Information that accompanies this paper.
