# Supplementary material for: Finding and analysing the minimum set of driver nodes required to control multilayer networks
Source: Sci Rep. 2019 Jan 24;9:576. doi: 10.1038/s41598-018-37046-z (PMC6345816; doi:10.1038/s41598-018-37046-z)
Supplement: Supplementary file 1 — Supplementary Information File [file 41598_2018_37046_MOESM1_ESM.pdf]

Supplementary Information File: Finding and analysing the minimum set of driver nodes required to control multilayer networks

Jose C. Nacher*, Masayuki Ishitsuka,
Department of Information Science, Faculty of Science,
Toho University, Funabashi, Chiba 274-8510, Japan

Shuichi Miyazaki
Academic Center for Computing and Media Studies,
Kyoto University, Kyoto 606-8501, Japan

Tatsuya Akutsu*
Bioinformatics Center, Institute for Chemical Research,
Kyoto University, Uji, 611-0011, Japan

1 Analysis of the Size of MDSM

In this section, we analyze the size of MDS for multilayer networks (i.e., the size of MDSM).

1.1 Formal Definition of MDSM

Recall that for a graph $G(V, E)$, U $(U \subseteq V)$ is called a *dominating set* (DS) of G if

$$(\forall v \in V)(v \in U \vee (\exists u \in U)(\{u, v\} \in E))$$

holds. A DS with the minimum cardinality is called a *minimum dominating set* (MDS).

MDSM is defined by extending this definition of MDS. Let $\mathcal{G} = \{G_i(V_i, E_i) | i = 1, \ldots, N\}$ be a set (multiset) of undirected networks. That is, $\mathcal{G}$ corresponds to multilayer networks. Let $V = \bigcup_{i \in \{1,\ldots,N\}} V_i$. U $(U \subseteq V)$ is called a *dominating set for multilayer networks* $\mathcal{G}$ (DSM for $\mathcal{G}$) if

$$(\forall i \in \{1, \ldots, N\})(\forall v \in V_i)(v \in U \vee (\exists u \in U)(\{u, v\} \in E_i))$$

holds. A DSM with the minimum cardinality is called a *minimum dominating set for multilayer networks* (MDSM).

1.2 Simple Upper and Lower Bounds

Let $V_{MDS}(G_i)$ be an MDS for G_i. Then, $S_{MDS}(G_i)$ denotes the MDS size for G_i (i.e., $S_{MDS}(G_i) = |V_{MDS}(G_i)|$). Let $V_{MDS}(\mathcal{G})$ and $S_{MDS}(\mathcal{G})$ denote an MDSM and its size for $\mathcal{G}$, respectively.

*JCN and TA are corresponding authors

Clearly, $S_{MDS}(\mathcal{G}) \geq S_{MDS}(G_i)$ holds for each G_i because an MDSM is also a DS for G_i. Conversely, $\bigcup_{i \in \{1,\ldots,N\}} V_{MDS}(G_i)$ becomes a DSM for $\mathcal{G}$. Therefore, we have the following.

Proposition 1 $\max_{i \in \{1,\ldots,N\}}\{S_{MDS}(G_i)\} \leq S_{MDS}(\mathcal{G}) \leq \sum_{i=1}^{N} S_{MDS}(G_i)$.

Although this result is almost obvious, it gives tight bounds in the worst case:

- if G_is are identical, $S_{MDS}(G_i) = S_{MDS}(\mathcal{G})$ holds for all G_i,
- if V_is are disjoint, $S_{MDS}(\mathcal{G}) = \sum_{i=1}^{N} S_{MDS}(G_i)$ holds.

This fact suggests that we should consider special family of networks in order to derive meaningful bounds. Furthermore, to be discussed later, results of computational experiments suggest that the size of MDSM for k-regular random networks is much smaller than this upper bound. In the following subsections, we theoretically explain this empirical finding.

1.3 Estimation of MDSM Size for k-Regular Random Networks

We assume that graphs are given uniformly at random on the same set of nodes V with $|V| = n$ under the constraint that every node has degree k for a constant k. We utilize the recursive probabilistic estimation technique that was recently developed for analysis of the size of an MDS in k-regular random networks and maximally assortative scale-free networks [5]. It is shown in [5] that this technique yields very accurate estimates of the MDS size for random graphs. It is to be noted that this technique does not give rigorous analysis methods but gives approximate analysis methods as done in many studies on complex networks using mean-field approximation and other approximate analysis techniques.

Let $G(U)$ denote the subgraph of G that is induced by a set of vertices U, and let $N_G(U)$ denote the set of neighbors of U in G excluding U (i.e., $N_G(U) = \{v | \{u,v\} \in E, u \in U, v \notin U\}$). We consider the following virtual procedure that outputs a DSM for $\mathcal{G} = \{G_1, \ldots, G_N\}$.

(i) Let DS_1 be the dominating set for G_1 obtained by the method in [5]. Let $V_1 \leftarrow V$.

(ii) For $i = 2$ to N, do steps (iii)-(iv).

(iii) Let $V_i \leftarrow V - \bigcup_{j=1}^{i-1} DS_j - N_{G_i}(\bigcup_{j=1}^{i-1} DS_j)$.
(V_i is the set of vertices in G_i that are not dominated by a combined dominating set for $G_1, \ldots, G_{i-1}$.)

(iv) Let DS_i be the dominating set for $G_i(V_i)$ obtained by the method in [5].

(v) Output $DS_1 \cup DS_2 \cup \cdots \cup DS_N$ as a DSM.

We estimate the size of DSM obtained by this procedure. Although DSM obtained by this procedure is not necessarily the minimum DSM, it is expected that the size of DSM gives an approximate upper bound of the size of MDSM.

As shown in [5], the size of DS_1 is estimated as

$$|DS_1| \approx \frac{n}{k+1}.$$

Then, the size of $N_{G_2}(DS_1)$ is estimated as

$$|N_{G_2}(DS_1)| \approx \left(n - \frac{n}{k+1}\right) \cdot \left[1 - \left(1 - \frac{1}{k+1}\right)^k\right]$$

because, in G_2, each vertex in $V - DS_1$ is connected to at least one vertex in DS_1 with probability $\approx 1 - (1 - \frac{|DS_1|}{|V|})^k$. Note that since G_2 is a random k-regular graph, DS_1 can be regarded as a random subset for G_2. Therefore, the size of V_2 is estimated as

$$|V_2| \approx \left(n - \frac{n}{k+1}\right) \cdot \left(1 - \frac{1}{k+1}\right)^k \; = \; n\left(1 - \frac{1}{k+1}\right)^{k+1}.$$

Accordingly, the size of DS_2 is estimated as

$$|DS_2| = \frac{n}{k+1}\left(1 - \frac{1}{k+1}\right)^{k+1}.$$

Here, we let

$$\alpha_i n = |\bigcup_{j=1}^{i} DS_j|.$$

For example, α_1 and α_2 are estimated as $\alpha_1 = \frac{1}{k+1}$ and $\alpha_2 = \frac{1}{k+1} + \frac{1}{k+1}\left(1 - \frac{1}{k+1}\right)^{k+1}$. By repeatedly applying the above argument, we have the following estimates:

$$\begin{aligned} |V_{i+1}| &= n(1-\alpha_i)^{k+1}, \\ \alpha_{i+1} &= \alpha_i + \frac{1}{k+1}(1-\alpha_i)^{k+1}. \end{aligned}$$

It seems difficult to obtain a simple analytical form of α_i. Therefore, we computed $\alpha_N n$ and compared them with the sizes of MDSM for randomly generated k-regular networks. Table S4 suggests that theoretical estimates are very accurate.

1.4 Estimation of MDSM Size for Maximally Assortative Scale-Free Networks

The above estimation method can be modified for analysis of maximally assortative scale-free networks in which the degree distribution follows a power-law $\propto k^{-\gamma}$. A network is called *maximally assortative* if exchange of any pair of edges does not increase the assortative coefficient [5]. Note that the assortative coefficient r for network $G(V,E)$ is given by

$$r \; = \; \frac{\left(\frac{1}{M}\sum_{e \in E} e_i e_j\right) - \left(\frac{1}{M}\sum_{e \in E}\frac{1}{2}(e_i + e_j)\right)^2}{\left(\frac{1}{M}\sum_{e \in E} e_i^2 + e_j^2\right) - \left(\frac{1}{M}\sum_{e \in E}\frac{1}{2}(e_i + e_j)\right)^2},$$

where e_i and e_j denote the degrees of endpoints of an edge e, and $M = |E|$.

In order to approximately analyze an upper bound of MDSM size, we employ the same virtual procedure as in Section 1.3. It is shown in [5] that a maximally assortative network is approximately regarded as a collection of k-regular networks. By using this property, as a base case, an upper bound of the MDS size ratio for a single maximally assortative network is estimated as

$$\beta_1 = \frac{\sum_{k=1}^{\infty}\frac{1}{k+1} \cdot k^{-\gamma}}{\sum_{k=1}^{\infty} k^{-\gamma}}$$

since there exist $\propto k^{-\gamma}$ nodes with degree k.

Assume that β_i has already been obtained as a size of MDSM DS for i networks. Then, there exist $(1-\beta_i)n$ vertices in G_{i+1} that do not belong to DS_i. The probability that a node v with degree k in $V - DS_i$ is not dominated by DS_i is estimated as

$$(1-\beta_i)^k,$$

because each edge from v is connected to a node in DS_i with probability β_i. Therefore, the expected number of nodes with degree k in $V - DS_i - N_{G_{i+1}}(DS_i)$ is estimated as

$$(1-\beta_i)n\left(\frac{((1-\beta_i)^k)k^{-\gamma}}{\sum_{k=1}^{\infty} k^{-\gamma}}\right).$$

Here we assume that $V - DS_i - N_{G_{i+1}}(DS_i)$ is a maximally assortative power-law network where the original degrees are preserved (we ignore the effect of degree change due to removal of edges to $N_{G_{i+1}}(DS_i)$). Since the size of MDSM is upper bounded by the size of $|DS_i \cup MDS(G_{i+1}(V - DS_i - N_{G_{i+1}}(DS_i)))|$, an upper bound of the MDSM size ratio for $i+1$ network is estimated as

$$\beta_{i+1} = \beta_i \ + \ \left(\frac{\sum_{k=1}^{\infty}(\frac{1}{k+1})(1-\beta_i)^{k+1}k^{-\gamma}}{\sum_{k=1}^{\infty} k^{-\gamma}}\right).$$

Again, it seems difficult to obtain a simple analytical form of β_i. Therefore, we computed $\beta_N n$ and compared them with the sizes of MDSM for artificially generated maximally assortative networks. It is seen from Table S5 that theoretical estimates are larger than MDSM sizes of artificially generated networks but both have similar tendencies. This result seems reasonable because this analysis gives estimates of upper bounds.

1.5 Convergence of MDSM Size for Multilayer Networks

Upper bound estimations and the result of computational experiments suggest that the MDSM size converges to n as the number of networks grows. We show that this speculation is true as shown below.

Proposition 2 *Suppose that each graph in multilayer networks has the minimum degree d_{min}. Then, the MDSM size is at least $n - d_{min}$ if a sufficient number of distinct graphs are given and n is sufficiently large.*

(Proof) Since any number of graphs can be given and n is sufficiently large (e.g., $n \geq 2d_{min}$ for d-regular graphs), we can assume w.l.o.g. that every set of $d_{min}+1$ nodes constitutes a star as an induced subgraph of some input graph.

Now we prove the proposition by contradiction. Suppose that there exists an MDSM DS with size less than $n - d_{min}$. Then, there must exist $d_{min}+1$ nodes $v_{i_1}, \ldots, v_{i_{d_{min}+1}}$ any of which does not belong to DS. From the assumption, the star centered at v_{i_1} with leaves $v_{i_2}, \ldots, v_{i_{d_{min}+1}}$ appears as an induced subgraph of some input graph G_j. However, v_{i_1} is not dominated by any of its neighbors in G_j. This contradicts to the assumption. □

2 Hardness of Computation of MDSM

It is known that the maximum bipartite matching for up to two-layer networks can be solved in polynomial time, whereas computation of such a matching for three or more layer networks is NP-hard [6]. This fact suggests that the minimum set of driver nodes under linear structural controllability [3] can be obtained in polynomial time only up to two-layer networks.

On the other hand, it is known that the computation of an MDS is NP-hard even for one network [7]. However, the situation changes if we consider special graph classes: it is known that MDS can be computed in polynomial time if a given network is a partial k-tree (for a constant k) [7]. As a special case, it is seen that an MDS can be computed in polynomial time if networks are forests or consist of cycles and stars. We show that computation of MDSM is NP-hard even for such simple networks.

Theorem 3 *The MDSM problem for two-layer networks is NP-hard even if a graph in each layer consists of cycles and at most one star.*

(Proof) We use a reduction from a special case of 3-SAT in which each variable occurs at most three times, at least once as a positive literal and at least once as a negative literal (see also Fig. S5). It is known that this special case remains NP-hard [8]. Let $\{x_1, \ldots, x_n\}$ and $\{c_1, \ldots, c_m\}$ be sets of variables and clauses, respectively, in an instance of this restricted 3-SAT.

We construct $G_1(V_1, E_1)$ by

$$\begin{aligned} V_1 &= \{v_0\} \cup \{v_i^1, v_i^2, v_i^3, v_i^4, v_i^5, v_i^6 |\ i \in \{1, \ldots, n\}\}, \\ E_1 &= \{\{v_i^j, v_i^{(j \bmod 6)+1}\}|\ i \in \{1, \ldots, n\}, j \in \{1, \ldots, 6\}\}. \end{aligned}$$

For each literal in a clause $c_j = \ell_{j_1} \vee \ell_{j_2} \vee \ell_{j_3}$, we define $v(\ell_{j_k})$ by

$$v(\ell_{j_k}) = \begin{cases} v_{j_k}^1, & \text{if } l_{j_k} \text{ is positive and the first occurrence of } x_{j_k}, \\ v_{j_k}^4, & \text{if } l_{j_k} \text{ is positive and the second occurrence of } x_{j_k}, \\ v_{j_k}^2, & \text{if } l_{j_k} \text{ is negative and the first occurrence of } \overline{x_{j_k}}, \\ v_{j_k}^5, & \text{if } l_{j_k} \text{ is negative and the second occurrence of } \overline{x_{j_k}}. \end{cases}$$

We construct $G_2(V_2, E_2)$ by

$$\begin{aligned} V_2 &= \{v_0\} \cup \{v_i^1, v_i^2, v_i^4, v_i^5 |\ i \in \{1, \ldots, n\}\}, \\ E_0 &= \{\{v(\ell_{j_1}), v(\ell_{j_2})\}, \{v(\ell_{j_2}), v(\ell_{j_3})\}, \{v(\ell_{j_3}), v(\ell_{j_1})\}|\ j \in \{1, \ldots, m\}\}, \\ E_2 &= E_0 \cup \{\{v_0, v_i^k\}|\ v_i^k \text{ does not appear in } E_0\}. \end{aligned}$$

The reduction can be done in polynomial time. We prove the correctness of this reduction. First, observe that an MDSM must contain v_0, and either $\{v_i^1, v_i^4\}$ or $\{v_i^2, v_i^5\}$ for each $i \in \{1, \ldots, n\}$. Therefore, the size of an MDSM is at least $2n + 1$. Next, suppose that the given 3-SAT instance is satisfied by an assignment $x_i = b_i$ ($i = 1, \ldots, n$, $b_i \in \{0, 1\}$). We begin with $S = \{v_0\}$. If $b_i = 1$, we add v_i^1 and v_i^4 to S. Otherwise (i.e., $b_i = 0$), we add v_i^2 and v_i^3 to S. Then, the resulting S is clearly a dominating set for both G_1 and G_2, and its size is $2n + 1$. Conversely, suppose that (G_1, G_2) has an MDSM of size $2n + 1$. As mentioned above, it must contain v_0, and either $\{v_i^1, v_i^4\}$ or $\{v_i^2, v_i^5\}$ for each $i \in \{1, \ldots, n\}$. If $\{v_i^1, v_i^4\}$ is contained, we let $x_i = 1$. Otherwise, we let $x_i = 0$. Then, the resulting assignment clearly satisfies all clauses. Therefore, the given 3-SAT instance is satisfiable iff (G_1, G_2) has an MDSM of size $2n + 1$. □

Theorem 4 *The MDSM problem for three-layer networks is NP-hard even if a graph in each layer does not contain any cycle.*

(Proof) We use a reduction from the same special case of 3-SAT as in the proof of Theorem 3 (see also Fig. S6).

We construct $G_1(V_1, E_1)$ by

$$\begin{aligned} V_1 &= \{v_i^1, v_i^2, v_i^3, v_i^4 |\ i \in \{1, \ldots, n\}\}, \\ E_1 &= \{\{v_i^1, v_i^2\}, \{v_i^3, v_i^4\}|\ i \in \{1, \ldots, n\}\}. \end{aligned}$$

We construct $G_2(V_2, E_2)$ by

$$\begin{aligned} V_2 &= \{v_i^1, v_i^2, v_i^3, v_i^4 |\ i \in \{1, \ldots, n\}\}, \\ E_2 &= \{\{v_i^1, v_i^4\}, \{v_i^2, v_i^3\}|\ i \in \{1, \ldots, n\}\}. \end{aligned}$$

For each literal in a clause $c_j = \ell_{j_1} \vee \ell_{j_2} \vee \ell_{j_3}$, we define $v(\ell_{j_k})$ by

$$v(\ell_{j_k}) = \begin{cases} v^1_{j_k}, & \text{if } l_{j_k} \text{ is positive and the first occurrence of } x_{j_k}, \\ v^3_{j_k}, & \text{if } l_{j_k} \text{ is positive and the second occurrence of } x_{j_k}, \\ v^2_{j_k}, & \text{if } l_{j_k} \text{ is negative and the first occurrence of } \overline{x_{j_k}}, \\ v^4_{j_k}, & \text{if } l_{j_k} \text{ is negative and the second occurrence of } \overline{x_{j_k}}. \end{cases}$$

We construct $G_3(V_3, E_3)$ by

$$\begin{aligned} V_3 &= \{c_j | j \in \{1, \ldots, m\}\} \ \cup \ \{v^h_i, u^h_i, w^h_i | \ i \in \{1, \ldots, n\}, \ h \in \{1, 2, 3\}\}, \\ E_3 &= \{\{c_j, v(\ell_{j_k})\} | \ j \in \{1, \ldots, m\}, \ k \in \{1, 2, 3\}\} \\ &\quad \cup \{\{v^h_i, u^h_i\}, \{u^h_i, w^h_i\} | \ i \in \{1, \ldots, n\}, \ h \in \{1, 2, 3\}\}. \end{aligned}$$

The reduction can be done in polynomial time. We prove the correctness of this reduction. First, observe that an MDSM must contain either $\{v^1_i, v^3_i\}$ or $\{v^2_i, v^4_i\}$ for each $i \in \{1, \ldots, n\}$, from which it follows that the size of an MDSM is at least $6n$. Next, suppose that the given 3-SAT instance is satisfied by an assignment $x_i = b_i$ ($i = 1, \ldots, n$, $b_i \in \{0, 1\}$). We begin with $S = \{\}$. If $b_i = 1$, we add $v^1_i, v^3_i, u^2_i, u^4_i, w^1_i$, and w^3_i to S. Otherwise (i.e., $b_i = 0$), we add $v^2_i, v^4_i, u^1_i, u^3_i, w^2_i$, and w^4_i to S. Then, the resulting S is clearly a dominating set for each G_i ($i = 1, \ldots, 3$), and its size is $6n$. Conversely, suppose that (G_1, G_2, G_3) has an MDSM of size $6n$. As mentioned above, it must contain either $\{v^1_i, v^3_i\}$ or $\{v^2_i, v^4_i\}$ for each $i \in \{1, \ldots, n\}$. If $\{v^1_i, v^3_i\}$ is contained, we let $x_i = 1$. Otherwise, we let $x_i = 0$. Then, the resulting assignment clearly satisfies all clauses. Therefore, the given 3-SAT instance is satisfiable iff (G_1, G_2, G_3) has an MDSM of size $6n$. □

The complexity of the MDSM problem of this special case for two layer networks is open.

3 Supplementary Information Tables (Table S1-S3 are given as Excel files)

Table S1 The results of the analysis of 70 metabolic networks from the angiosperm plant lineage including eudicot and monocot subgroups, the early land plant and green algae lineages. (Upper matrix) The number of common enzymes between both species are shown by $|V|$. MDSM and MDSI numbers are indicated in each box cell. (Lower matrix) The network size and the computational time (milliseconds) required to execute the FAST-MDSM method are shown. The total number of nodes and the MDS for each single network are shown in the first column.

Table S2 Same as Table S1 but considering a 6-layer network analysis in which each group consist of up to three species. This leads to a set of 25 groups for the 6-layer analysis.

Table S3 Same as Table S1 but for protein-protein interaction networks corresponding to five organisms and constructed from HINT database version 4.0. The results in upper matrix show that the size of the MDSM is close to that of the MDSI (see 2-layer result for *H.sapiens* vs. *D.Melanogaster*). Computational time is small in spite of the large size of the networks. The file also shows results for the 3-layer and 4-layer and protein networks.

Table S4 Comparison of theoretical estimates and computational results on the size of MDSM for multilayer k-regular random networks, where $n = 50$ and the average size over 5 trials is shown for each of the computational results.

Table S5 Comparison of theoretical estimates and computational results on the size of MDSM for multilayer power-law networks, where $n = 100$ and the average size over 5 trials is shown for each of the computational results.

4 Supplementary Information Figure Captions

Fig. S1: Illustration of rewiring operations, where black circles represent nodes in V_{MDS}. In case (i), v_j or v_h needs to be added to V_{MDS} unless v_j has another neighbour in V_{MDS}. In case (ii), v_j or v_h needs to be added to V_{MDS} unless either v_j or v_h has another neighbour in V_{MDS}.

Fig. S2: Sizes of the MDSM and MDS for scale-free graphs of $n \approx 5000$ when random rewiring operations are applied, where K denotes the number of random rewiring operations and the cases of $K = 0$ correspond to the MDS.

Fig. S3: (a) Comparison of the actual and estimated ratios of the size of the MDSM to that of the MDS when random rewiring operations are applied. (b) Comparison of the actual and estimated ratios of the size of MDSM to that of the MDS when the random deletion and insertion of edges are applied. Empty and filled nodes indicate simulation results and theoretical predictions, respectively.

Fig. S4: Same as Fig. 2 (main text) but for protein-protein interaction networks corresponding to five organisms and constructed from the HINT database version 4.0. The results shown in Fig. S4 (upper matrix) reveal that the size of the MDSM is close to that of the MDSI (see *H.sapiens* vs. *D.Melanogaster* results). Computational time is small in spite of the large size of the networks.

Fig. S5: Example of a reduction in the proof of Theorem 3 for 3-SAT instance of $\{x_1 \vee \overline{x_2} \vee x_3, x_1 \vee x_2 \vee \overline{x_3}\}$. Black edges and red edges represent those in G_1 and G_2, respectively.

Fig. S6: Example of a reduction in the proof of Theorem 4 for 3-SAT instance of $\{x_1 \vee \overline{x_2} \vee x_3, x_1 \vee x_2 \vee \overline{x_3}\}$. Black, blue, and red edges represent those in G_1, G_2, and G_3, respectively.

Fig. S7. Graphs G_1 and G_2 can be combined into a single graph $G_1 \cup G_2$ as shown in figure. In these examples, all three graphs lead to the same $|MDS|$=3 (filled nodes).

Fig. S8. Computational results of the MDS(G_1), MDS(G_2) and MDS($G_1 \cup G_2$) using eight plant metabolic networks extracted from four different lineages (eudicots: red, monocots: blue, early land plants: yellow and green algae: green).

References

[1] Maki, Y., Tominaga, D., Okamoto, M., Watanabe, S. & Eguchi, Y. 2001 Development of a system for the inference of large scale genetic networks. *Proc. Pacific Symp. Biocomputing* **6**, 446-458 (2001).

[2] Mochizuki, A., Fiedler. B., Kurosawa, G. & Saito, D. Dynamics and control at feedback vertex sets. II: A faithful monitor to determine the diversity of molecular activities in regulatory networks. *Journal of Theoretical Biology* **335**, 130-146 (2013).

[3] Liu, Y.-Y., Slotine, J.-J. & Barabási, A.-L. Controllability of complex networks. *Nature* **473**, 167-173 (2011).

[4] Nacher, J.C. & Akutsu, T. Dominating scale-free networks with variable scaling exponent: heterogeneous networks are not difficult to control. *New Journal of Physics* **14**, 073005 (2012).

[5] Takemoto, K. & Akutsu, T. Analysis of the effect of degree correlation on the size of minimum dominating sets in complex networks. *PLoS ONE* **11**, e0157868 (1984).

[6] Bredereck, R., Komusiewicz, C., Kratsch, S. Molter, H., Niedermeier, R., & Sorge, M. Assessing the computational complexity of multi-layer subgraph detection. *Lecture Notes in Computer Science* **10236**, 128-139 (2017).

[7] Fomin, F.V. & Kratsch, D. *Exact Exponential Algorithms.* Springer: Berlin, 2010.

[8] Garey, M.R. & Johnson, D.S. *Computers and Intractability. A Guide to the Theory of NP-Completeness.* W. H. Freemand and Company: NY, 1979.

Tables S4-S5 (Tables S1-S3 are given as Excel files)

	theoretical				computational			
N	$k=3$	$k=4$	$k=5$	$k=6$	$k=3$	$k=4$	$k=5$	$k=6$
1	12.5	10.0	8.3	7.1	13.8	11.4	10.0	9.0
2	16.4	13.2	11.1	9.5	16.6	14.2	12.0	11.0
3	18.9	15.4	12.9	11.1	18.2	16.0	14.0	12.2
4	20.8	16.9	14.3	12.3	20.2	17.2	15.0	13.4
5	22.2	18.2	15.4	13.3	21.6	18.0	16.0	14.0
6	23.4	19.2	16.3	14.1	22.4	19.0	16.6	14.8
7	24.4	20.1	17.1	14.8	23.0	19.4	17.0	15.0
8	25.3	20.9	17,7	15.4	23.8	20.2	17.8	16.0
9	26.0	21.5	18.3	16.0	25.0	21.0	18.0	16.0

Table S4

	theoretical			computational		
N	$\gamma=2.5$	$\gamma=3$	$\gamma=4$	$\gamma=2.5$	$\gamma=3$	$\gamma=4$
1	43.9	46.3	48.5	40.4	45.2	48.2
2	56.5	58.9	61.0	44.8	47.4	49.4
3	64.0	66.2	68.1	50.4	52.6	54.8
4	69.0	71.1	72.9	54.8	56.6	58.6
5	72.7	74.6	76.3	58.0	60.2	62.2
6	75.6	77.3	78.9	60.8	63.4	64.8
7	77.9	79.5	81.0	63.0	65.2	67.2
8	79.7	81.3	82.7	65.0	67.4	69.2
9	81.3	82.8	84.0	67.0	68.8	70.6

Table S5

Supplementary Material Figures S1-S7

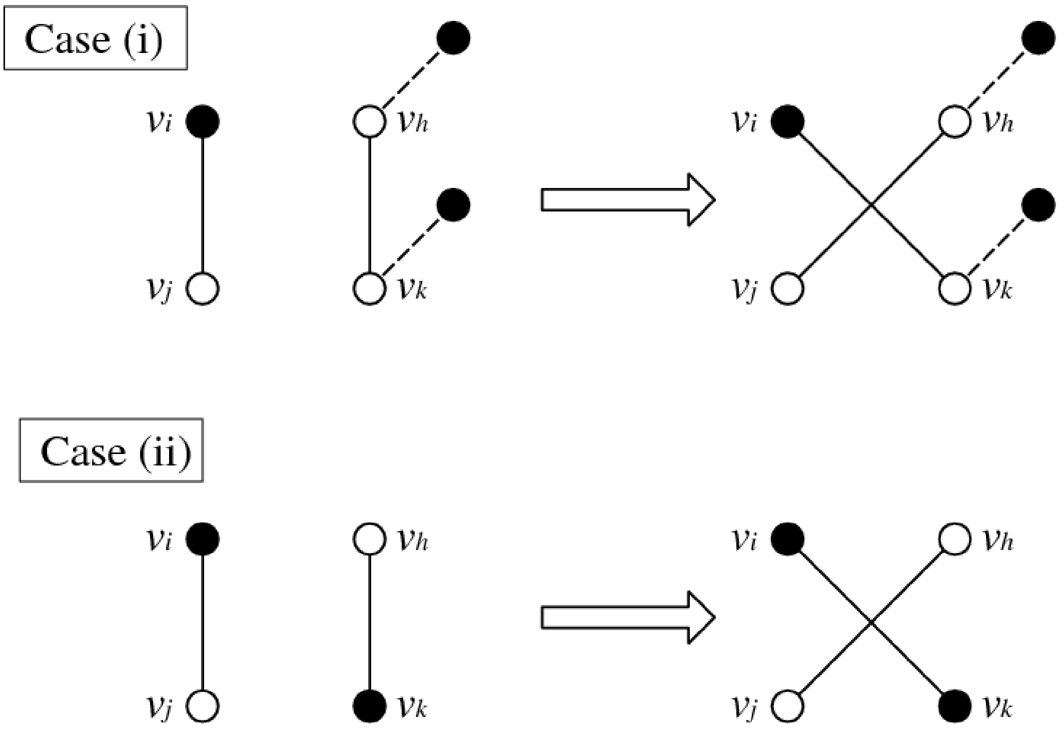

Fig. S1

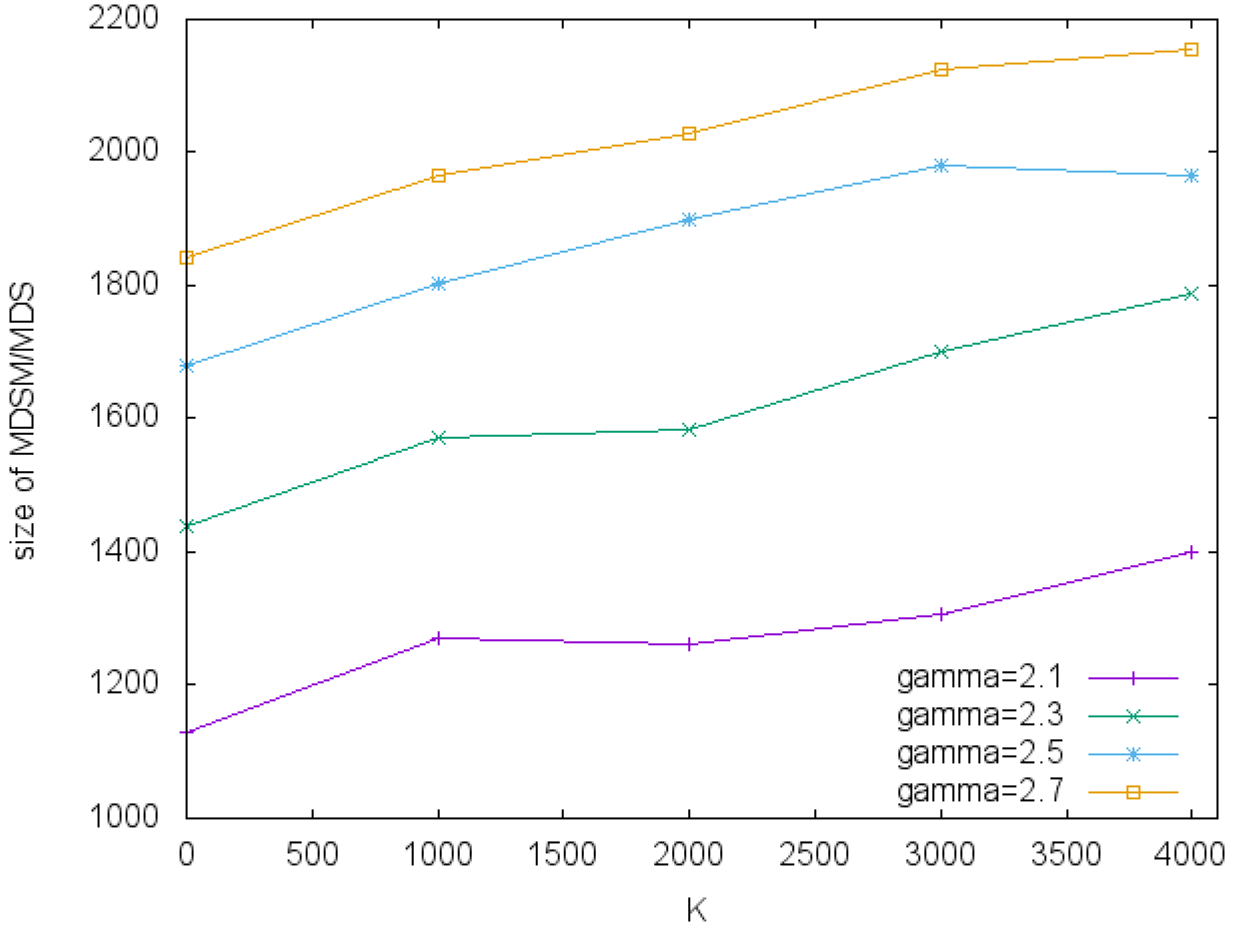
2200
2000
1800
1600
1400
1200
1000
size of MDSMMDS
0
500
1000
1500
2000
2500
3000
3500
4000
K
gamma=2.1
gamma=2.3
gamma=2.5
gamma=2.7

Fig. S2

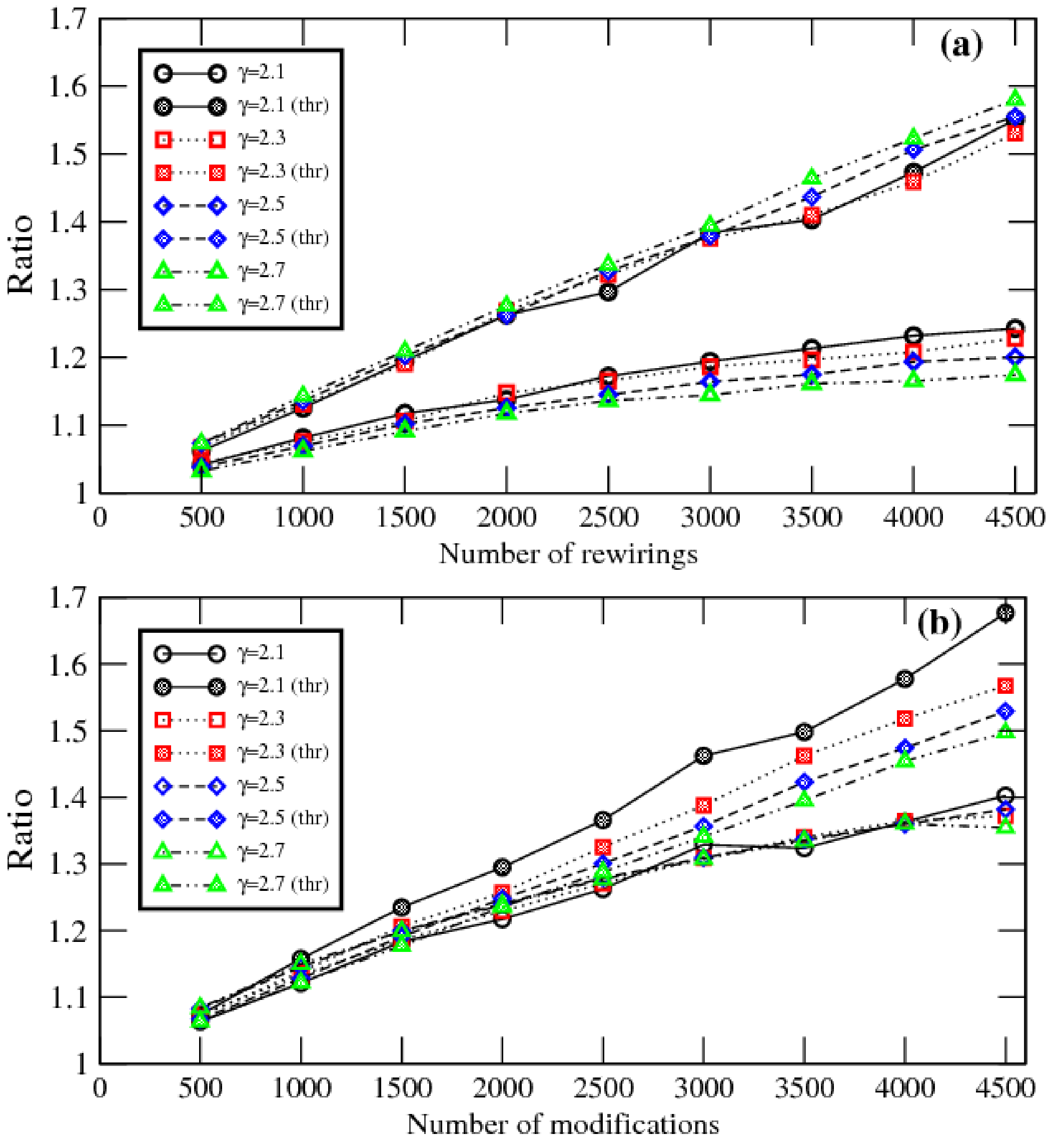

(a)
γ=2.1
γ=2.1 (thr)
γ=2.3
γ=2.3 (thr)
γ=2.5
γ=2.5 (thr)
γ=2.7
γ=2.7 (thr)
Ratio
Number of rewirings
(b)
γ=2.1
γ=2.1 (thr)
γ=2.3
γ=2.3 (thr)
γ=2.5
γ=2.5 (thr)
γ=2.7
γ=2.7 (thr)
Ratio
Number of modifications

Fig. S3

<table>
<tr><th>#Node
#MDS</th><th>HINTv4 – Binary, Main component
(KEY : Gene name)</th><th>H. sapiens</th><th>D. melanogaster</th><th>S. cereviciae</th><th>A. thaliana</th><th>C. elegans</th></tr>
<tr><td>11711
1714</td><td>Homo sapiens</td><td></td><td>Overlap : |V| 581
MDSM 289
MDSI 244</td><td>Overlap : |V| 454
MDSM 241
MDSI 198</td><td>Overlap : |V| 168
MDSM 127
MDSI 121</td><td>Overlap : |V| 3
MDSM 3
MDSI 3</td></tr>
<tr><td>7422
1438</td><td>Drosophila melanogaster</td><td>Network Size 18552
Computational Time
131.3 ms</td><td></td><td>Overlap : |V| 336
MDSM 190
MDSI 165</td><td>Overlap : |V| 100
MDSM 82
MDSI 81</td><td>Overlap : |V| 18
MDSM 18
MDSI 18</td></tr>
<tr><td>5146
851</td><td>Saccharomyces cerevisiae S288C</td><td>Network Size 16403
Computational Time
102.1 ms</td><td>Network Size 12232
Computational Time
100.5 ms</td><td></td><td>Overlap : |V| 197
MDSM 137
MDSI 131</td><td>Overlap : |V| 0
MDSM 0
MDSI 0</td></tr>
<tr><td>4839
841</td><td>Arabidopsis thaliana</td><td>Network Size 16382
Computational Time
71.5 ms</td><td>Network Size 12161
Computational Time
60.7 ms</td><td>Network Size 9788
Computational Time
73.2 ms</td><td></td><td>Overlap : |V| 1
MDSM 1
MDSI 1</td></tr>
<tr><td>4292
791</td><td>Caenorhabditis elegans</td><td>Network Size 16000
Computational Time
69.1 ms</td><td>Network Size 11696
Computational Time
66.8 ms</td><td>Network Size 9438
Computational Time
– ms</td><td>Network Size 9130
Computational Time
71.8 ms</td><td></td></tr>
</table>

Fig. S4

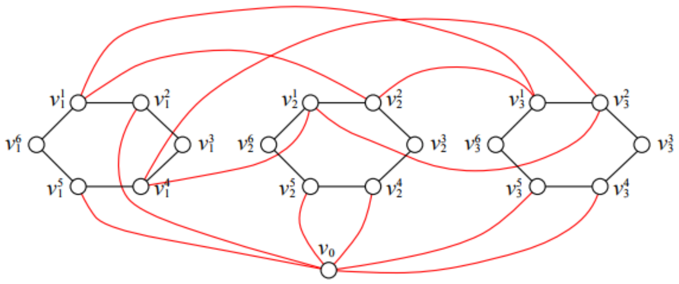

Fig. S5

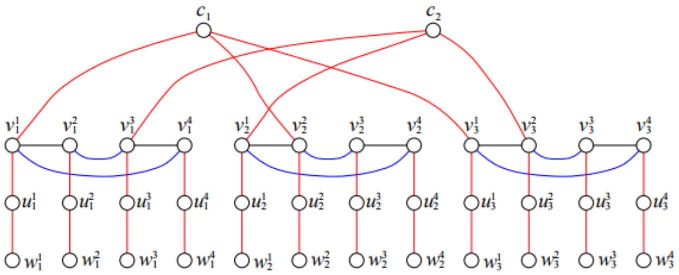

Fig. S6

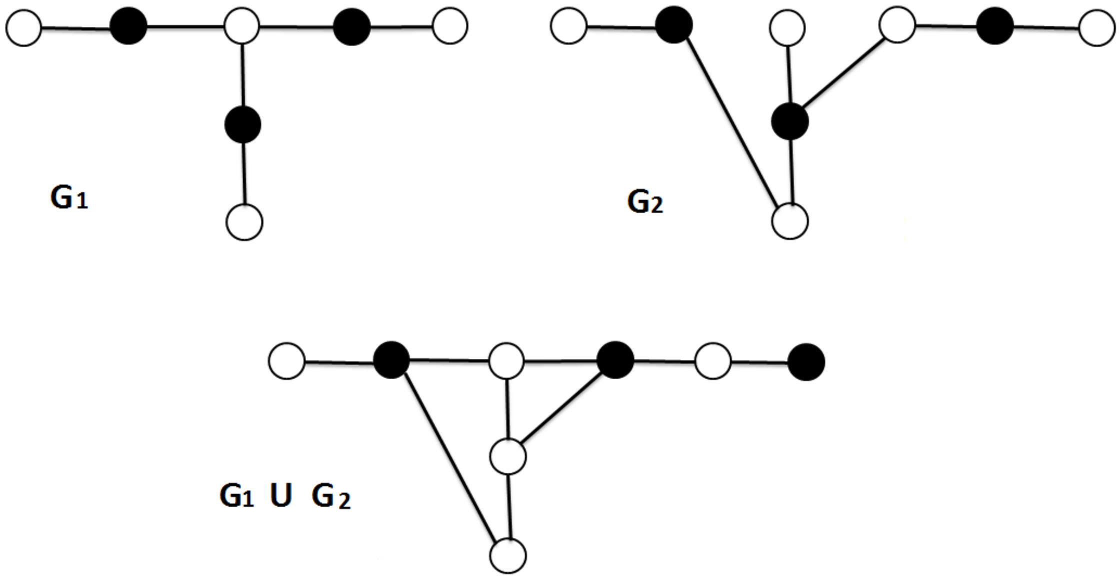

Fig. S7

Plantcyc - Main component<br>G1 \ G2	boleracea_capitatacyc	brapa_fpsccyc	oglaberrimacyc	oryzacyc	mosscyc	selaginellacyc	csubellipsoideacyc	mpusillacyc
Brassica oleracea var. capitata (boleracea_capitatacyc)		MDS(G1) 289<br>MDS(G2) 279<br>MDS(G1∪G2) 294	MDS(G1) 289<br>MDS(G2) 281<br>MDS(G1∪G2) 327	MDS(G1) 289<br>MDS(G2) 284<br>MDS(G1∪G2) 322	MDS(G1) 289<br>MDS(G2) 276<br>MDS(G1∪G2) 340	MDS(G1) 289<br>MDS(G2) 261<br>MDS(G1∪G2) 310	MDS(G1) 289<br>MDS(G2) 226<br>MDS(G1∪G2) 314	MDS(G1) 289<br>MDS(G2) 208<br>MDS(G1∪G2) 311
Brassica rapa FPsc (brapa_fpsccyc)			MDS(G1) 279<br>MDS(G2) 281<br>MDS(G1∪G2) 316	MDS(G1) 279<br>MDS(G2) 284<br>MDS(G1∪G2) 312	MDS(G1) 279<br>MDS(G2) 276<br>MDS(G1∪G2) 328	MDS(G1) 279<br>MDS(G2) 261<br>MDS(G1∪G2) 302	MDS(G1) 279<br>MDS(G2) 226<br>MDS(G1∪G2) 306	MDS(G1) 279<br>MDS(G2) 208<br>MDS(G1∪G2) 304
Oryza glaberrima (oglaberrimacyc)				MDS(G1) 281<br>MDS(G2) 284<br>MDS(G1∪G2) 297	MDS(G1) 281<br>MDS(G2) 276<br>MDS(G1∪G2) 323	MDS(G1) 281<br>MDS(G2) 261<br>MDS(G1∪G2) 299	MDS(G1) 281<br>MDS(G2) 226<br>MDS(G1∪G2) 306	MDS(G1) 281<br>MDS(G2) 208<br>MDS(G1∪G2) 301
Oryza sativa japonica group (oryzacyc)					MDS(G1) 284<br>MDS(G2) 276<br>MDS(G1∪G2) 323	MDS(G1) 284<br>MDS(G2) 261<br>MDS(G1∪G2) 301	MDS(G1) 284<br>MDS(G2) 226<br>MDS(G1∪G2) 307	MDS(G1) 284<br>MDS(G2) 208<br>MDS(G1∪G2) 306
Physcomitrella patens (mosscyc)						MDS(G1) 276<br>MDS(G2) 261<br>MDS(G1∪G2) 301	MDS(G1) 276<br>MDS(G2) 226<br>MDS(G1∪G2) 296	MDS(G1) 276<br>MDS(G2) 208<br>MDS(G1∪G2) 298
Selaginella moellendorffii (selaginellacyc)							MDS(G1) 261<br>MDS(G2) 226<br>MDS(G1∪G2) 292	MDS(G1) 261<br>MDS(G2) 208<br>MDS(G1∪G2) 281
Coccomyxa subellipsoidea C-169 (csubellipsoideacyc)								MDS(G1) 226<br>MDS(G2) 208<br>MDS(G1∪G2) 251
Micromonas pusilla CCMP1545 (mpusillacyc)								

Fig. S8
